# Olfactory Receptor Multigene Family in Vertebrates: From the Viewpoint of Evolutionary Genomics

**DOI:** 10.2174/138920212799860706

**Published:** 2012-04

**Authors:** Yoshihito Niimura

**Affiliations:** Department of Bioinformatics, Medical Research Institute, Tokyo Medical and Dental University, 1-5-45 Yushima, Bunkyo-ku, Tokyo 113-8510, Japan

**Keywords:** Birth-and-death evolution, Color vision, G-protein coupled receptor, Multigene family, Odor perception, Olfactory receptor, Primate evolution, Vertebrate evolution.

## Abstract

Olfaction is essential for the survival of animals. Diverse odor molecules in the environment are detected by the olfactory receptors (ORs) in the olfactory epithelium of the nasal cavity. There are ~400 and ~1,000 OR genes in the human and mouse genomes, respectively, forming the largest multigene family in mammals. The relationships between ORs and odorants are multiple-to-multiple, which allows for discriminating almost unlimited number of different odorants by a combination of ORs. However, the OR-ligand relationships are still largely unknown, and predicting the quality of odor from its molecular structure is unsuccessful.

Extensive bioinformatic analyses using the whole genomes of various organisms revealed a great variation in number of OR genes among species, reflecting the diversity of their living environments. For example, higher primates equipped with a well-developed vision system and dolphins that are secondarily adapted to the aquatic life have considerably smaller numbers of OR genes than most of other mammals do. OR genes are characterized by extremely frequent gene duplications and losses. The OR gene repertories are also diverse among human individuals, explaining the diversity of odor perception such as the specific anosmia.

OR genes are present in all vertebrates. The number of OR genes is smaller in teleost fishes than in mammals, while the diversity is higher in the former than the latter. Because the genome of amphioxus, the most basal chordate species, harbors vertebrate-like OR genes, the origin of OR genes can be traced back to the common ancestor of the phylum Chordata.

## INTRODUCTION

There are a variety of odors in our environment. If the molecule of β-phenylethyl alcohol reaches at the olfactory epithelium of your nose, you recognize it as a fragrance of rose. On the other hand, the amyl acetate molecule is perceived as the odor of bananas.

The olfactory system can well be understood by being compared with the color vision system. Light is defined as electromagnetic radiation visible to the human eye and has wavelengths between 380 and 780 nm. Light of a single wavelength has a characteristic color among spectral colors. As Isaac Newton pointed out that “The rays are not coloured” in the 17th century, color is a subjective experience and is not a physical property of light. For example, if red light and green light enter the eye simultaneously, the resulting color perceived in the brain is pure yellow, which is indistinguishable from single-wavelength yellow light, though the physical properties of these lights are totally different. Moreover, a mixture of red and blue lights produces magenta, an extra-spectral color.

All colors perceived by humans are represented by an appropriate mixture of three primary colors, red, green, and blue. The physiological basis of the presence of the primary colors is that we humans (except for color-blind people) have three different classes of cone cells in the retina, each of which contains a different photopigment showing a distinctive spectral sensitivity. A photopigment is composed of the protein opsin and the light-sensitive 11-*cis* retinal, and each of the three photopigments contains a different opsin.

**Fig. (1):**
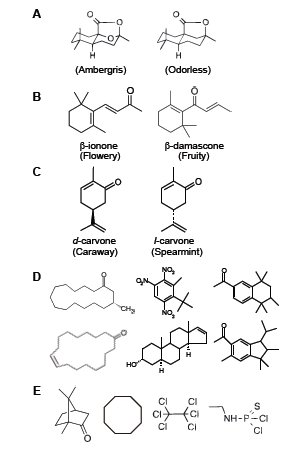
Complicated structure-odor relationships [1, 2]. (**A**) Molecules that are similar in structure showing different odors. (**B**) Molecules that have identical functional groups smell differently. (**C**) Enantiomeric pairs showing different odors. (**D**) Diverse molecules having musk odors. (**E**) Diverse molecules having camphoraceous odors.

In the case of color vision, when the wavelength of a light stimulus changes continuously, the perceived color also changes gradually. This is usually not true for olfaction. For instance, the left molecule in Fig. (**1A**) have a strong ambergris odor, whereas its carbon analog (right) is odorless [1]. Functional groups roughly determine the quality of odors; *e.g.*, a thiol group (-SH) gives strong unpleasant odors like garlic or rotten eggs, while esters smell fruity. However, solely the presence of functional groups cannot explain the odor of molecules. For example, β-ionone (Fig. **1B**, left) has a fragrance of violet flowers, whereas β-damascone (right) shows a fresh fruity smell. Fig. (**1C**) indicates an example of enantiomeric pairs (mirror images) having different smells [2]: *d*-carvone (left) smells like caraway, while *l*-carvone (right) smells like spearmint.

Conversely, molecules that are completely different in structure often exhibit similar odors. All of the diverse molecules illustrated in Fig. (**1D**) have musk odors [2]. Fig. (**1E**) is another example: Although there are no chemical functionalities that are common to the four molecules, they show camphoraceous odors that are similar to each other. The only common structural features of them are their molecular shape and size: All of them are roughly spherical or egg-shaped with a diameter of ~7Å [1].

As seen in Fig. (**1**), the structure-odor relationships are complicated [1], and the quality of odor is generally unpredictable from its molecular structure. Even now, creation of a new fragrance by perfumers is a matter of trial and error. The relationships among stimuli, receptors, and perception are still poorly understood for olfaction. One of the reasons for the complexity of olfactory perception is due to a large number of receptors. Humans have ~400 different receptors for detecting odors, which is in sharp contrast to the case of color vision medicated by only three opsins. This also explains the reason why the search for ‘primary odors’, from which all of the perceivable odors could be reproduced by appropriate mixtures, has been unsuccessful.

Among the five senses, olfaction is often regarded as the least important sense for humans. However, olfaction is essential for the survival of most animals, and it is used for finding foods, avoiding dangers, identifying mates and offspring, marking territory, etc. Mice and cows, for example, have >1,000 different receptors for odor detection. Therefore, apparently most mammals live in a much richer olfactory world than humans do. In fact, mice [3] and flies [4] have a receptor for detecting CO_2_, which is odorless to humans.

Olfactory receptors were first identified from rats by Linda Buck and Richard Axel in 1991 [5]. They found a huge multigene family of G-protein coupled receptors (GPCRs) of which expression is restricted to the olfactory epithelium (OE). They then estimated the number of OR genes in the mammalian genomes to be ~1,000. This estimation was proven to be correct, when the whole genomes of various mammals became available. Since mammalian genomes harbor 20,000–25,000 genes, 4–5% of the entire proteome is ORs.

Buck and Axel’s seminal paper opened the door to the molecular studies of chemosensation. During the following decade, many other chemosensory receptor genes – taste and pheromone receptors and ORs from various organisms – were also identified [6, 7]. 

Because ORs are the sensor to the external world, by examining the repertoires of OR genes among diverse organisms, we can infer how do genomes respond to the environmental changes. Moreover, it is intriguing to investigate the evolutionary dynamics of OR genes, because they form an enormous multigene family (the OR multigene family is regarded to be the largest multigene family in vertebrates). In this review, I would summarize our knowledge on the evolutionary dynamics of OR multigene family from the viewpoint of comparative genomics.

## EXPRESSION

Volatile odorants that enter the nasal cavity and dissolve in the nasal mucus are detected by ORs on the cilia of olfactory sensory neurons. Olfactory neurons are embedded in the OE, a small patch in humans covering a region of ~5cm^2^ in the back of the nasal cavity. These neurons directly project axons to the olfactory bulb of the brain. Olfactory neurons are exceptionally exposed to the exterior to directly access to odorant molecules, and as a consequence they are subject to continual damage from potentially harmful substances in the air such as airborne pollutants, allergens, or microorganisms. For this reason, olfactory neurons are continuously replaced to new ones during 30–60 days.

It is generally thought that each olfactory neuron expresses only a single functional OR gene from one of the two alleles out of ~1,000 genes [8, 9]. This ‘one neuron–one receptor rule’ is thought to be important for olfactory coding, such that only a given population of olfactory neurons responds to a restricted number of odor molecules. Despite immense efforts, however, the molecular mechanisms regulating the activation of one OR allele remain elusive. It was suggested that one functional OR gene is stochastically chosen in each olfactory neuron and its expression prevents the activation of other OR genes through negative feedback regulation [10–12]. Lomvardas *et al*. [13] reported that a single enhancer termed the H region, which derives from the homology between mouse and human [10], acts in *trans* to activate only one of the multiple OR promoters on different chromosomes. However, genetic ablation of the H region disrupts the expression of only three proximal ORs, suggesting that the H region rather functions in *cis* [14, 15]. More recently, another model was proposed [16]. According to this model, all of the OR alleles become silenced before OR transcription, and later a stochastically chosen allele is transcriptionally activated. The synthesis of OR protein elicits a feedback signal to prevent from desilencing another allele and stabilize the chosen allele.

OR genes are predominantly expressed in the OE, but it was reported that a small subset of OR genes were expressed in the vomeronasal organ in mice [17]. The vomeronasal organ is involved in pheromone detection in many mammals, though it is absent in humans. Previously the vomeronasal organ was regarded to be specialized for detecting pheromones, but now the main olfactory system and the vomeronasal system are assumed to have some overlapping functions [18].

Some OR genes are expressed in testis, and these ORs are apparently involved in sperm chemotaxis [19]. In addition, several researches reported that OR genes are expressed in various non-olfactory tissues including the tongue, brain, kidney, placenta, etc. However, the significance of such ‘ectopic’ expressions is unclear in most cases. Feldmesser *et al*. [20] found no correlations in expression levels in non-olfactory tissues by comparing orthologous OR genes between humans and mice, suggesting that ectopically expressed OR genes are merely ‘transcriptional noises’. On the contrary, De la Cruz *et al*. [21] detected a statistically significant correlation for human-chimpanzee OR orthologs. This result implies that ectopically expressed OR genes are under evolutionary constraint and thus ORs may have some additional functions in non-olfactory tissues.

## STRUCTURE OF OR PROTEINS

ORs are members of GPCRs, receptors that associate with a G-protein for their signal transduction after the activation by extracellular ligands. GPCRs have a common structural feature having seven α-helical transmembrane (TM) regions. They can be classified into five or six groups by sequence similarities, and OR genes belong to a rhodopsin-like GPCR superfamily, which is the largest one among them. This superfamily includes opsin genes for detecting lights and many other receptor genes for neurotransmitters, peptide hormones, chemokines, lipids, nucleotides, etc. [22].

**Fig. (2):**
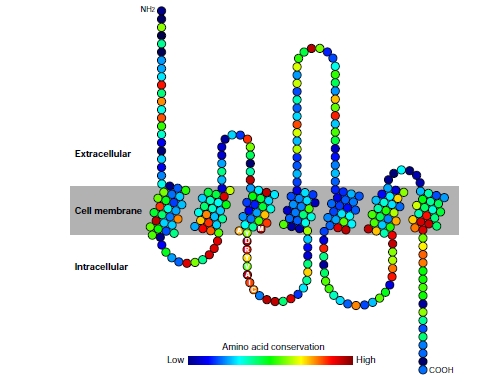
Structure of an OR. The extent of conservation at each amino acid position is shown by a color in the color chart, with blue being highly variable and red being highly conservative. The position of ‘MAYDRYVAIC’ motif is also shown.

ORs are ~310 amino acids long on average. There are several motifs characteristic of ORs. One such motif is ‘MAYDRYVAIC’ located at the junction of TM3 and the intracellular loop between TM3 and TM4 (Fig. **2**). Within this motif, the stretch of three amino acids, ‘DRY’ (aspartic acid–arginine–tyrosine), is highly conserved among rhodopsin-like GPCRs. The DRY motif is possibly important for G-protein coupling, though the precise mechanism of G-protein activation is still unclear [23]. Multiple alignment of numerous mammalian ORs indicates that the extent of conservation is relatively low at TM3 to TM6 (Fig. **2**), suggesting that these regions are involved in ligand binding. 

Man *et al*. [24] identified 22 putative ligand-binding sites using human and mouse ORs by assuming that amino acid positions interacting with specific odorants would be conserved between orthologs but variable among paralogs. A majority of these positions are located in TM3 to TM7, which are predicted to form a binding pocket of ORs. We should note that, however, the assumption may not be true for all ORs. Zhuang *et al*. [25] reported that the ligand sensitivity and/or specificity can dramatically differ even in orthologous ORs among higher primates.

Because of the lack of a high-resolution OR structure, exact amino acid positions for recognizing and discriminating odorants are largely unknown. There are several studies in which computational and experimental approaches are combined to elucidate the ligand-binding sites of particular ORs. Katada *et al*. [26] conducted rhodopsin-based homology modeling and ligand docking simulation studies together with functional analysis and site-directed mutagenesis to identify ligand-binding site of the mouse eugenol receptor (mOR-EG). The study revealed nine amino acid positions located in TM3, TM5, and TM6, four of which overlap with those by Man *et al*. [24]. Recently Baud *et al*. [27] performed more detailed analyses for the mOR-EG and identified 11 amino acid sites, 10 of which are in TM3 to TM6 and one in the extracellular loop between TM2 and TM3. Moreover, ligand-binding amino acid positions were investigated by using similar approaches for the mouse receptor of dicarboxylic acids [28] and human receptor of citronellic terpenoid [29], suggesting the positions in TM3, TM5, and TM6, and in TM2 to TM7, respectively.

## LIGANDS

It is generally thought that an OR detects a part of an odorant molecule, such as a functional group, rather than the entire shape of a molecule. Therefore, one OR recognizes multiple odorants with related structures, while one odorant is recognized by multiple ORs. In other words, the relationships between odorants and ORs are multiple-to-multiple, and different odorants are recognized as different combinations of ORs. This scheme is called the ‘combinatorial coding’ [30]. Since the number of possible combination of ORs is immense, this scheme could allow for the discrimination of an almost unlimited number of different odorants.

Some odorants are perceived as different odors at different concentrations. A striking example is skatole, which has a flowery smell at a low concentration, whereas of which smell is an unpleasant fecal odor at a high concentration. (Skatole is naturally found in essential oils of jasmine or orange blossoms as well as mammalian feces.) This phenomenon is explained by the combinatorial coding scheme, because the same odorant could be represented as different combinations of ORs depending on its concentration, given that the threshold for the activation of ORs are variable.

Saito *et al*. [31] performed high-throughput screening of 93 diverse odorants against 464 human and mouse ORs that are expressed in heterologous cells. Their results showed that the combinatorial coding scheme is indeed correct. Moreover, it was demonstrated that some ORs are ‘generalists’, which bind to a variety of ligands, whereas others are ‘specialists’ that are narrowly tuned to a small number of ligands.

Note that, however, they could identify ligands for 52 mouse and 10 human ORs out of 464 ORs examined [31]. Therefore, currently our knowledge of OR-ligand relationships is still limited. Moreover, it is unknown which part of an odorant molecule is recognized by each OR. As mentioned above, the relationships between odorant molecules and odor qualities are complicated, and to understand the ‘odor code’, it is necessary to investigate a larger number of OR-ligand relationships.

## OR GENES AND GENOMIC DISTRIBUTION

OR genes do not have any introns in their coding regions. However, an OR gene often has additional 5´ untranslated exons at the upstream of the coding region. These non-coding exons can be alternatively spliced to generate multiple mRNA isoforms, which, however, results in the same protein [32]. Therefore, the significance of the alternative splicing of OR genes is unclear. The intronless gene structure is not specific to OR genes, but is widely observed among rhodopsin-like GPCR genes [33].

OR genes form genomic clusters and are dispersed in many chromosomes. Human OR genes are located on all chromosomes except chromosomes 20 and Y, and chromosome 11 contains >40% of them [34]. There are >30 genomic clusters containing five or more OR genes in the human genome. The largest human OR gene cluster contains ~100 OR genes (including pseudogenes) occupying an ~2Mb genomic region on the short arm of chromosome 11. OR genes are generally densely arrayed in a cluster without interspersed non-OR genes, and the distribution of intergenic distances between neighboring human OR genes shows a sharp peak at ~1.1 kilobase (kb) [34].

OR genes located close to each other within a cluster tend to be evolutionarily closely related, while duplication of the whole OR gene cluster appears to be rare [34]. It is therefore suggested that the number of OR genes has increased by repeating the following process: A gene duplication occurred *via *unequal crossing-over, and then mutations accumulated to diversify the sequences of the duplicates.

At the same time, however, one OR gene cluster often contains evolutionarily distantly related genes, and OR genes with a close evolutionary relationship are located at different clusters or chromosomes [34]. These observations can be explained by assuming that several chromosomal rearrangements have occurred at the regions of OR gene clusters and the genes in different clusters were shuffled. In fact, it is known that the chromosome fission event that generated human chromosomes 14 and 15 had occurred at a cluster of OR genes in the common ancestor of great apes [35]. Moreover, it has been reported that a reciprocal translocation is medicated by OR gene clusters on chromosomes 4 and 8, causing a genetic disease Wolf-Hirschhorn syndrome [36].

The organization of OR gene clusters is generally well conserved between humans and mice. Although mice have a much larger number of OR genes than humans do, the numbers of gene clusters containing five or more OR genes are nearly the same between the two species [37]. Therefore, on average, each mouse cluster contains a larger number of OR genes than a human cluster. The largest mouse cluster contains >270 OR genes and spans a >5Mb region.

## GENE REPERTOIRES AND EVOLUTION

### Identification of OR Genes

Since the complete genome sequence of a bacterium *Haemophilus influenzae* was decoded in 1995, the whole genomes of diverse organisms have been sequenced. As is well known, the draft assembly of the human genome sequence became available in 2001, and the completion of the far more accurate finished sequence of the human genome was declared in 2003. Recently, the appearance of next-generation DNA sequencers made it possible to produce sequencing data much faster and much more cheaply, and determination of the complete sequences of 1,000 human individuals is in progress.

Several groups including myself have identified the entire set of OR genes from various organisms by using bioinformatic methods. Fig. (**3**) summarizes the numbers of OR genes found from the whole genome sequences of 27 chordate species. As shown in this figure, the numbers of OR genes are highly variable among different species. In general, the fraction of OR pseudogenes is high (20–60%), which is characteristic of the OR gene family.

In Fig. (**3**), OR genes identified were classified into three categories, ‘intact genes’, ‘truncated genes’, and ‘pseudogenes’. An intact gene is defined as a continuous sequence that starts from the initiation codon and ends with the stop codon without any long deletions and that contains well-conserved motifs. On the other hand, a pseudogene is a sequence containing nonsense mutations, frameshifts, and/or long deletions. A truncated gene represents a partial sequence of an intact gene. Some genomic data include a large number of fragmental sequences of several kb or several tens of kb because of incomplete sequence assembly. In such cases, only a part of an OR gene sequence is found from the database. The presence of large numbers of truncated genes in mouse lemur, bush baby, and tree shrew is because their genome sequences are at a low coverage (<2x). 

Truncated genes may become intact if the genome sequencing is completed. (It is also possible that a truncated gene is actually a pseudogene.) For this reason, here I calculated the fraction of pseudogenes for each species by assuming that both intact and truncated genes are functional.

Note that, however, even an intact gene might actually be nonfunctional. The reason is that it will take some time to accumulate nonsense and/or frameshift mutations within a coding region after a gene has lost its function. For example, occurrence of deleterious mutations in a promoter region may preclude the expression of a gene, though its coding region still remains intact.

Menashe *et al*. [38] proposed to use more conservative criteria to discriminate functional OR genes from pseudogenes. Their algorithm is based on deviations from a functionally crucial consensus, consisting of sixty highly conserved positions identified by a comparison of two evolutionarily constrained OR repertories in mice and dogs with a small pseudogene fraction. According to this algorithm, ~35% of the human OR genes having intact coding regions were likely to be inactive, while such OR genes in mice are only ~5%. This observation is consistent with the fact that OR genes have been degenerated during the evolutionary process of higher primates (see below).

Zhang *et al*. [39] extensively examined the expression of OR gene in the human OE by using a custom-made microarray. In this study, they assumed that the expression of a given OR gene is detected when its expression level is significantly elevated in the OE compared to other five tissues (testis, lung, kidney, heart, and liver). The results demonstrated that 80% of intact OR genes are indeed expressed in the OE of humans. However, interestingly, 67% of the OR pseudogenes with one or two disruptive mutations also showed a significant expression in the OE. This is probably because of a regulatory mechanism specific of OR gene expression, which may allow OR pseudogenes to be transcribed into mRNAs. Therefore, the distinction between functional OR genes and OR pseudogenes by experiments is not straightforward. To confirm the functionality of a given OR gene, it would be necessary to confirm its translation into a protein.

### Human

In 2003, we reported 388 intact OR genes and 414 OR pseudogenes identified from the complete human genome sequence [34], while the numbers changed into 396 and 425 for intact genes and pseudogenes, respectively, in 2010 due to the update of the human genome sequence [40]. More than half (52%) of the entire set of human OR genes are pseudogenes. The slight modification of numbers is not essential, however, because the exact number of OR genes in the human genome cannot be uniquely determined as we see below.

OR gene loci are known to be one of the most genetically diverse regions in the human genome. Recent studies suggest that the human genome contains thousands of deletions or duplications of DNA segments greater than 1kb in size, which is present in some individuals but not in others. Such variation of genome structure is called copy number variation (CNV). Several studies reported that OR genes are significantly enriched in the CNV regions [41–44], suggesting that a given OR gene loci is missing in some individuals. OR gene coding regions also harbor a large number of single nucleotide polymorphisms (SNPs), some of which lead to inactivation of OR genes (*i.e.*, segregating pseudogenes). According to the analyses using 150 individual genomes, ~15% and ~20% of functional OR genes are affected by CNVs and segregating pseudogenes, respectively [44]. Therefore, the numbers of OR genes vary among individuals.

It is also known that olfactory perception largely differs among individuals. There is a phenomenon called specific anosmia, in which certain individuals having a generally good sense of smell nevertheless lack the ability to perceive the odor of a specific substance [45]. For example, one in ten individuals cannot perceive the extremely poisonous gas, hydrogen cyanide. (This molecule has a faint almond-like odor for most people.) Moreover, one among 1,000 does not smell butyl mercaptan, which is the odor of skunks and is artificially added to natural gas.

Androstenone, a pig pheromone, is another example of specific anosmia. There are three different types of perception for this molecule: offensive (sweaty, urinous), pleasant (sweet, floral), and odorless. Keller *et al*. [46] demonstrated that the perception of androstenone is associated with the polymorphism residing in an OR gene named *OR7D4*, and the receptor OR7D4 is activated by androstenone *in vivo*. There are two non-synonymous SNPs linked to each other, R88W and T133M in this gene locus. Subjects having *RT*/*WM* or *WM*/*WM* genotypes were less sensitive and felt less unpleasant to androstenone than *RT*/*RT* subjects. The *OR7D4* genotype explained 19% and 39% of the variance in pleasantness and intensity of the steroid odors, respectively. The remaining variance can be due to other OR genotypes, other genes, or non-genetic factors such as cultural influences.

Menashe *et al*. [47] reported the association between a segregating OR pseudogene *OR11H7P* and sensitivity of a sweaty odorant, isovaleric acid. The study showed that hypersensitivity towards this odorant (hyperosmia, an opposite phenomenon to anosmia) is predominantly found in individuals carrying at least one intact allele of *OR11H7P*.

There are a number of studies claiming that some OR genes are under positive selection in the human lineage [25, 48–53]. Several sequence analyses of a genome-wide scan suggested a significant enrichment of chemosensory receptor genes with evidence for positive selection in humans [49–51, 53]. In these studies, human-specific adaptive changes were inferred by identifying genes with accelerated evolutionary rates compared with expectation. Recently, however, the validity of these approaches to detect positively selected genes has been challenged, because biased gene conversion can also account for the accelerated evolution [54, 55].

Moreno-Estrada *et al*. [52] showed that a human OR gene, *OR5I1*, is under positive selection by conducting population genetic analyses based on 39 human populations. On the other hand, Gimelbrant *et al*. [56] found no evidence of positive selection on the human OR gene repertoire as a whole. Moreover, Zhuang *et al*. [25] conducted functional analyses of a human OR gene, *OR7D4*, and pointed out that the vast majority of amino acid sites causing functional changes were not detected by a computational method. Therefore, the extent of positive selection affecting human OR genes is controversial. As we will see below, OR genes in the human and other primate lineages has been degenerated; thus, the contribution of positive selection to the evolution of the human OR gene repertoire, if any, would be minor.

### Primates

As shown in Fig. (**3**), primates tend to have smaller numbers of OR genes compared with other mammals. This observation reflects the fact that primates heavily rely on vision rather than olfaction and thus their olfactory ability has been retrogressed. In fact, the relative size of olfactory bulb in the brain and that of OE in the nasal cavity are smaller in primates than in most other mammals [57].

The order Primates are classified into two suborders, the strepsirrhines (lemurs and lorises), meaning ‘curved nose’, and the haplorhines (tarsiers, New World monkeys (NWMs), Old World monkeys (OWMs), and hominoids (apes and human)), meaning ‘simple nose’, based on the shape of their nostrils. This classification is supported by molecular studies [58]. Strepsirrhines and haplorhines are characterized by the presence and absence of the rhinarium, respectively. The rhinarium is the moist and hairless surface at the tip of the nose and is used to detect the direction of odorants. It is present in many mammals such as cats and dogs.

**Fig. (3):**
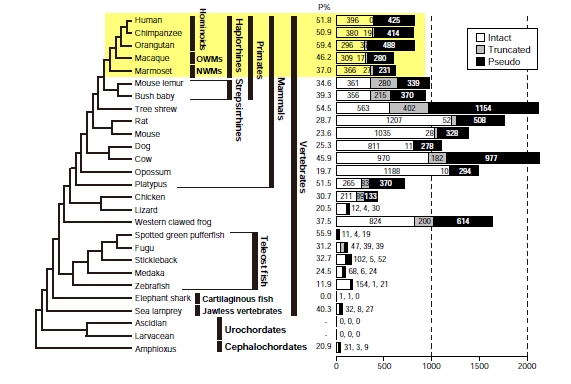
Numbers of OR genes identified from the whole genome sequences of various organisms [28, 43, 49]. The numbers in the bar graphs indicate those of intact genes, truncated genes, and pseudogenes. The fraction of pseudogenes (P%) in each species was computed from the number of pseudogenes divided by the total number of OR genes. Here we assume that all of the intact and truncated genes are functional for the calculation of P%. However, because some truncated genes may actually be pseudogenes, P% is inaccurate for the species with a low-coverage genome (*i.e.*, the species having a large number of truncated genes). A yellow box indicates five haplorhine species with deep-coverage genomes (see the main text).

Haplorhines showed a smaller relative size of OE than strepsirrhines [59]. Moreover, most strepsirrhines are nocturnal while most haplorhines are diurnal, and color vision is well developed only in haplorhines (see below). Therefore, the reliance on olfaction is decreased in haplorhines compared with strepsirrhines. In consistent with these observations, the number of putatively functional OR genes (intact + truncated genes) is smaller in haplorhines than in strepsirrhines (Fig. **3**).

Several studies claimed that the degradation of OR genes has accelerated in the human lineage after the divergence from chimpanzees, and thus humans have accumulated more OR pseudogenes than chimpanzees [60–62]. However, our analyses using the deep-coverage (6x) chimpanzee genome demonstrated that the number of putatively functional OR genes and the fraction of pseudogenes are very similar between the two species [63]. Moreover, there are no significant differences in the rate of pseudogenization events and the number of genes under positive selection.

Although the numbers of putatively functional OR genes are almost the same between humans and chimpanzees, their OR gene repertories are considerably different. Our analyses suggested that ~25% of the repertories are specific to each species [63]. This observation can be explained by assuming that the most recent common ancestor (MRCA) of the two species had a large OR gene repertoire covering both human and chimpanzee repertories, but lineage-specific losses of OR genes occurred in each lineage after the divergence of the two species. This also implies that the spectrum of perceivable odorants may be quite different between them.

Mammals are dichromatic (color-blindness) in general, while OWMs and hominoids exceptionally possess complete trichromatic vision. As explained earlier, trichromatic vision is medicated by three different photopigments (opsins + retinals) having specific spectral sensitivities. Trichromacy in OWMs and hominoids became possible because the X-linked opsin gene was duplicated to generate red and green opsin genes in the OWMs/hominoids ancestral lineage. Color vision system in NWMs is special: The X-linked opsin gene locus is polymorphic in general, and thus heterozygous females are trichromatic, while homozygous females and all males are dichromatic.

To see a possible link between color vision and olfaction, Gilad *et al*. [61] investigated the fractions of OR pseudogenes from 19 primate species by examining 100 OR genes that were randomly chosen. The results showed that the fractions of OR pseudogenes in OWMs and hominoids are significantly higher than NWMs and other mammals. From these observations, they hypothesized that OR genes were lost concomitantly with the acquisition of complete trichromatic vision (‘color vision priority hypothesis’ [7, 40]). However, our whole-genome analyses [40] demonstrated that there are no significant differences between hominoids/OWMs and NWMs (Fig. **3**), which does not support the color vision priority hypothesis. Interestingly, orangutans and macaques have rather smaller repertoires of OR genes than humans. Therefore, in contrast to our intuition, the sense of smell in humans may not be inferior to other higher primates.

We also estimated the number of OR gene losses in each lineage of the primate evolution using the data from five primate species with deep-coverage genomes (indicated by a yellow box in Fig. **3**) [40]. The results showed a gradual loss of OR genes in every branch from the MRCA of the five species to the human rather than a sudden loss at the branch of the OWMs/hominoids ancestor, at which the acquisition of full trichromatic vision occurred. Therefore, this analysis again does not support the color vision priority hypothesis. One reason of the erroneous conclusion by Gilad *et al*. [61] might be that they postulated that the fractions of OR pseudogenes are anti-correlated with the numbers of functional OR genes. In reality, the number of pseudogenes can change easily in evolution, and therefore the fraction of OR pseudogenes is merely a poor indicator of the number of functional OR genes.

### Mammals

Mammalian OR genes are classified into Class I and Class II according to amino acid sequence similarity. In human and mouse functional OR genes, Class I genes are 15% and 11%, respectively [64]. The ligands to Class I and Class II ORs tend to be hydrophilic and hydrophobic, respectively, but detailed functional differences between the two classes are not yet understood [31]. Interestingly, both in the human and mouse genomes, all Class I OR genes are encoded in a single genomic cluster [37].

Many non-primate mammalian species have ~1,000 OR genes in their genomes, indicating the importance of olfaction for these species (Fig. **3**). However, dolphins (toothed whales), which secondarily adapted to the marine habitat, are exceptional and have completely lost the olfactory apparatus. They have no olfactory bulbs [65]. A nose (called a blowhole) is located at the top of a head, but it solely function for breathing. Vision is also ineffective in their underwater habitat impenetrable to light. Instead, dolphins have developed a sophisticated echolocation system. They emit clicking sounds, and then measure the time delay between their own sounds and echoes to produce three-dimensional images of their targets. Apparently the olfactory system that had been used in their terrestrial ancestors did not work in the aquatic life (see below). In fact, the number of intact OR genes in the dolphin genome is extremely small and almost all of the OR genes found are pseudogenes (Niimura, unpublished). As mentioned above, it takes some time to accumulate disruptive mutations in a coding region of a gene after the gene was inactivated; therefore, intact genes found from the dolphin genome are likely to be nonfunctional.

Platypuses also have a small OR gene repertoire (Fig. **3**). They are semi-aquatic egg-laying mammals endemic to Australia. The platypus bill is a sensor, a combination of electroreceptors and mechanoreceptors. It can detect weak electric fields generated by preys such as freshwater shrimps in the mud at the bottom of streams. Therefore, platypuses can find their preys with their eyes, ears, and nostrils closed.

As we have seen in the cases of primates, dolphins, and platypuses, different modalities of senses affect to each other. The OR gene repertoire existing in each organism’s genome reflects its living environment and the extent of reliance on olfaction. The analyses have shown that the OR gene family is characteristic of extremely frequent gene gains and losses [40, 63, 64, 66, 67]. This kind of a dynamic evolutionary process of multigene families is called birth-and-death evolution. It is known that any multigene families follow birth-and-death evolution to some extent, but the OR multigene family is one of the most extreme cases [7, 68].

Dogs are famous for their keen olfactory sense. Nevertheless, the number of OR genes in the dog genome is not particularly large (Fig. **3**). It can be thought that the number of OR genes in a given species are positively correlated with the number of odorants it can discriminate among, but the sensitivity to a specific odorant may be determined by absolute expression levels of OR genes. The different types of odors that carnivores need to distinguish may not be large, though this is merely a speculation at this moment.

### Vertebrates and Other Chordates

Odorants disperse more slowly in the water than in the air, nevertheless smell is an important source of information also for fish. Fish use olfactory cues for finding foods, avoiding danger, and identifying individuals as mammals do. In addition, olfactory information is used to recognize places in their environment. It is known that salmon have a remarkable homing ability to return to the river they were spawned in. This ability relies on olfaction: Salmon imprint to place-specific odors during a sensitive developmental period, and adults use the odorant memory to return to their natal streams.

Most fish have four nostrils. A paired olfactory organ is located in their snout, each of which consists of an olfactory cavity connecting with the exterior through two opening, one in and one out. Due to a one-way flow of water carrying odorants, they are constantly able to access new odor information. Fish detect mainly four groups of water-soluble molecules as odorants: amino acids, gonadal steroids, bile acids, and prostaglandins [69]. They are non-volatile chemicals and thus humans cannot smell them. As shown in Fig. (**3**), teleost fishes (zebrafish, medaka, stickleback, fugu, and spotted green pufferfish) generally have much smaller numbers of OR genes than mammals [70]. The variation in the number of functional OR genes is >10-fold among teleost fishes examined.

**Fig. (4):**
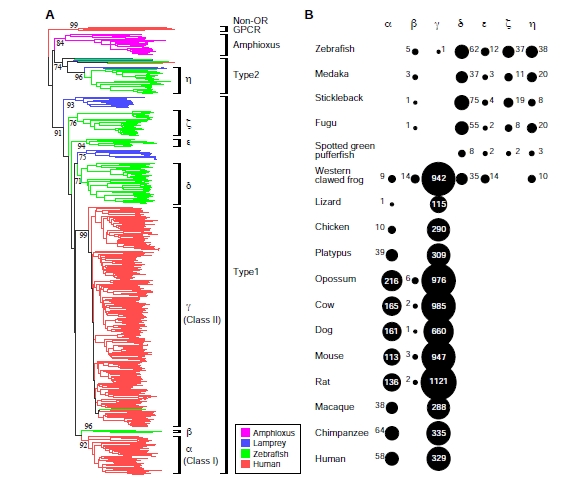
(**A**) Phylogenetic tree constructed by using all intact OR genes from amphioxus, lamprey, zebrafish, and human. Several non-OR GPCR genes were used as the outgroup. (**B**) Number of functional genes (the sum of intact genes and truncated genes) belonging to each group for each species. Modified from ref. [49].

Extensive phylogenetic analyses showed that vertebrate OR genes can be separated into Type 1 and Type 2 genes (Fig. **4A**) [66, 70]. As mentioned above, mammalian OR genes are classified into Class I and Class II, but both classes belong to Type 1. Lampreys belong to jawless vertebrates (agnathans), the earliest-diverging lineage in living vertebrates. Because both Type 1 and Type 2 clades contain lamprey OR genes, the divergence between Type 1 and Type 2 clades was more ancient than the divergence between jawless and jawed vertebrates. OR genes in teleost fishes and tetrapods (amphibians, reptiles, birds, and mammals) were classified into seven groups named α–η [66, 70]. Among the seven groups, α–ζ belong to Type 1, while group η is in Type 2 clade. Type 2 clade also contains some diverse genes in addition to group η genes, but they are apparently non-OR genes [70].

Fig. (**4B**) shows the numbers of functional OR genes belonging to groups α–η in each species. The distribution of genes suggests that these groups can be further classified into two categories. One category contains groups α and γ that are present in tetrapods but are absent in fish (with the exception of one intact gene in zebrafish). The other consists of groups δ, ε, ζ, and η, which were found in teleost fishes and amphibians, whereas reptiles, birds, and mammals completely lack them. Interestingly, only amphibians retain both categories of genes.

This observation indicates that the former category of genes is for detecting airborne odorants, while the latter is for water-soluble odorants [66, 70]. Group β genes are exceptional, because they were present both in aquatic and terrestrial vertebrates. It is therefore possible to speculate that group β genes detect odorants that are both water-soluble and airborne, such as alcohol [70], though there is no experimental evidence. In mammals, group γ corresponds to Class II, while group α and β correspond to Class I.

It is thought that the MRCA of teleost fishes and tetrapods retained a set of OR genes belonging to the seven groups. In the tetrapod lineage, however, all the genes but groups α and γ (and a small number of group β genes) had been lost during the process of terrestrial adaptation. Because the importance of olfaction is apparently greater in the land than in the water, the number of OR genes had enormously increased by repeated gene duplications in the tetrapod lineage [66, 70, 71]. For this reason, although fish have much smaller number of OR genes than mammals do, OR genes in fish are more diverse than those in mammals (Fig. **4A**).

The phylum Chordata consists of three subphyla, vertebrates, urochordates, and cephalochordates. Among the three subphyla, cephalochordates are at the most basal position in the Chordata phylogeny. Amphioxus (lancelet), a member of cephalochordates, is a peculiar organism also called a ‘headless animal’ (acraniate). It lacks any distinctive olfactory apparatus. Nevertheless, >30 vertebrate-type OR genes were identified from the genome sequences of the Florida lancelet [70]. Amphioxus OR genes are highly divergent from vertebrate OR genes, but they are clearly distinct from other non-OR GPCRs. They form amphioxus-specific clade in a phylogenetic tree and are characterized by long C-terminal tails. At least one of the genes identified is broadly expressed in bipolar neurons of the rostral epithelium of an adult amphioxus [72], but the olfactory system of amphioxus is still poorly understood and thus more detailed examination should be necessary.

By contrast, no OR-like genes were found from the genome sequences of urochordates. Absence of vertebrate-type OR genes in the urochordate genomes examined suggests that all OR genes were lost in their lineages. Ascidians are sessile filter-feeders, while larvaceans have a floating planktonic lifestyle. Reflecting their relatively inactive lifestyles, the nervous systems of urochordates are highly reduced and sensory receptors are poorly developed. However, the possibility that other families of genes function as chemosensory receptors in urochordates cannot be excluded.

OR genes were also identified from other invertebrates including insects, nematodes, echinoderms, and mollusks [71]. However, their evolutionary origins are distinct from that of vertebrate OR genes. The neuroanatomical features of insect and vertebrate olfactory systems are common, but insect and vertebrate OR genes are strikingly different to each other and share no sequence similarities [73]. Insect ORs are seven-transmembrane proteins, but their membrane topology is inverted compared with vertebrate ORs and other rhodopsin-like GPCRs [74]. Moreover, insect ORs are ligand-gated ion channels that function as heterodimers [75, 76]. Some nematode OR genes and echinoderm OR genes are members of rhodopsin-like GPCRs; however, they form lineage-specific clades that are distinct from the vertebrate OR gene clade in a phylogenetic tree of rhodopsin-like GPCRs [50]. It therefore appears that the genes encoding chemosensory receptors have evolved many times independently in animal evolution.

## CONCLUDING REMARKS

The discovery of OR genes in 1991 opened the door to the molecular studies of olfaction. During two decades after the discovery, our understanding of the olfactory system has become astonishingly deepened. Nevertheless, still many problems remain unsolved in the field of olfactory researches, such as the regulatory mechanism of the one neuron–one receptor rule, deorphanizing every OR in various organisms, or predicting the quality of odor from its molecular structure.

In this review I focused on the evolution of OR genes from the perspective of comparative genomics. Comparative studies among a broad range of species have demonstrated that the OR gene families are characterized by extremely frequent gene duplications and pseudogenizations, causing drastic changes in the number of genes during evolution. The outstanding examples are shrinking OR gene repertoires in higher primates that are equipped with well-developed visual sense and distinctive repertories between terrestrial and aquatic vertebrates. Apparently the OR genes reside in each species’ genome is largely affected by species-specific factors such as its habitat and diet. Availability of the whole-genome sequences from more diverse organisms will provide us deeper understanding to the evolution of OR gene families and to the interaction between genomes and environments in general.
